# The Emergence, Role, and Impact of Recovery Support Services

**DOI:** 10.35946/arcr.v41.1.04

**Published:** 2021-03-25

**Authors:** Leonard A. Jason, Meghan Salomon-Amend, Mayra Guerrero, Ted Bobak, Jack O’Brien, Arturo Soto-Nevarez

**Affiliations:** Center for Community Research, DePaul University, Chicago, Illinois

**Keywords:** recovery high schools, collegiate recovery programs, recovery homes, recovery coaches, recovery community centers, alcohol

## Abstract

Various community recovery support services help sustain positive behavior change for individuals with alcohol and drug use disorders. This article reviews the rationale, origins, emergence, prevalence, and empirical research on a variety of recovery support services in U.S. communities that may influence the likelihood of sustained recovery. The community recovery support services reviewed include recovery high schools, collegiate recovery programs, recovery homes, recovery coaches, and recovery community centers. Many individuals are not provided with the types of environmental supports needed to solidify and support their recovery, so there is a need for more research on who may be best suited for these services as well as when, why, and how they confer benefit.

Across the different developmental stages of the life course, alcohol and other drugs play an influential role in health, functioning, disease, disability, and premature mortality. A number of different approaches have emerged during the past 60 years to address areas impacted by alcohol and drug use, including formal professional treatment services, but also—in recognition of the need for ongoing support following acute care stabilization—a variety of recovery support services. This article reviews several recovery support services, including recovery high schools, collegiate recovery programs, recovery homes, recovery coaches, and recovery community centers. The article examines the role and implications of recovery support services across diverse subpopulations of individuals with alcohol or drug use disorders and related problems. It begins with a review of the prevalence rates and unmet needs for services across the life span for those with alcohol and drug use disorders.

According to recent national estimates, 17% of adolescents report using illicit drugs, and 5% engage in binge drinking.^1^ Additionally, 24% of full-time college students ages 18 to 22 report using illicit drugs, and 16% and 11% meet the diagnostic criteria for a drug use disorder or alcohol use disorder (AUD), respectively.^2^ At any given time, an estimated 4% of the college student population is in recovery from substance use disorder (SUD), including AUD.^3^ Students in recovery face many challenges when pursuing higher education, including exposure both to the high availability of alcohol or other drugs and to peers using substances on college campuses. These risk factors are further compounded by difficulties commonly experienced by students, including transitional stress and academic challenges, which can increase their susceptibility to engage in alcohol and drug use. Additionally, students who attend college full-time are more likely to consume considerable amounts of alcohol compared to their peers who are either not attending college or who are enrolled in college part-time.^4^ Many youth in high school and college settings are exposed to environments that encourage drug use experimentation, and few recovery programs are available and accessible.

Although 8% to 9% of the adult U.S. population has an alcohol or drug use problem at any given time, only 2% of the population seek and receive treatment each year for these disorders (about 3.8 million individuals),^5^ and even individuals who successfully complete treatment have high relapse rates. Posttreatment, individuals often live in communities that do not provide environmental recovery programs. However, there is an emerging network of recovery high schools, collegiate recovery programs, recovery housing, recovery coaches, and recovery community centers throughout the United States. This article’s goal is to engage in an integrative review that summarizes past literature to provide a comprehensive understanding of the rationale, origins, emergence, prevalence, and research associated with these recovery support services. The articles mentioned below were the result of MEDLINE, Google Scholar, and PsycINFO searches that included the following terms: recovery high schools, collegiate recovery programs, recovery housing, recovery coaches, and recovery community centers.

## RECOVERY HIGH SCHOOLS

Beginning in the late 1970s, recovery high schools (RHS) were established to serve youth recovering from drug use disorders.^6^ Currently, there are more than 35 RHS across the United States. Most of these schools have licensed counselors and staff to provide recovery support. Students are usually required to attend outside support groups, such as 12-step programs. According to Finch and colleagues, the enrollment range in RHS is between six and 50 students, with one to five teachers.^6^ Some RHS have independent physical structures and organizations, whereas others share space with public high schools. In addition, some RHS support students’ transition back to traditional high schools, whereas others retain students until graduation. The lack of steady referrals to RHS can pose challenges to remaining financially viable.

Tanner-Smith and colleagues explored the characteristics of students who attend RHS.^7^ In comparison to national samples, they found that students from RHS were significantly older, were more likely to be female and White, and reported higher levels of social support. In addition, RHS students were more likely to have family histories of drug use and mental health problems. Their parents also tended to have higher socioeconomic status than the general population. Compared to local non-RHS samples, students from RHS had higher rates of illicit drug use and drug use treatment episodes and fewer problems with illegal activities and arrests. In addition, students from RHS were more likely to suffer from other types of mental illness and to seek treatment alongside their drug use. Treatment centers appear to provide the majority of referrals, followed by family and self-referrals.

A few studies have evaluated the experiences and outcomes of those provided RHS. For example, Finch reported that the structure of RHS helped students maintain sobriety by separating them from traditional high school students, providing support groups comprising peers undergoing recovery, and making available staff with expertise in drug use recovery.^8^ In addition, students mentioned that RHS led to increases in abstinence self-efficacy. Karakos found that RHS staff felt that students received emotional support and information on peer-to-peer recovery, and that RHS students gained new social network members who replaced those who engaged in drug use.^9^ The small school sizes led to strong bonds as well as increased accountability because relapse was harder to hide. However, RHS staff also reported that students experienced peer pressure to engage in alcohol and drug use and risky behavior during social outings. In addition, staff had to help students navigate boundaries around sharing information about their sobriety on social media.

In one of the few outcome studies, Finch et al. compared alcohol and drug use and educational outcomes between students in RHS and those attending other high schools.^7^ They found that students attending RHS were more likely to be abstinent from alcohol and drugs and less likely to be absent from school than students in other high schools, but there were no significant differences in academic performance and mental health outcomes.

Oser et al. noted that youth of color often lack access to treatment prior to enrolling in RHS.^10^ Glaude et al. found high rates of drug use among Hispanic youth, yet they lack access to interventions tailored for them.^11^ RHS that include culturally specific elements may represent a promising setting for this population; however, additional research is warranted to determine the effectiveness of RHS among Hispanic youth.

## COLLEGIATE RECOVERY PROGRAMS

In response to the challenges students in recovery face, collegiate recovery programs (CRPs) have formed on college campuses nationwide to help students manage their recovery while completing their education. CRPs provide students with a network of peers in recovery and with institutional support in the form of services and academic guidance. The first CRP was developed in 1977 at Brown University, and there are now 138 active programs throughout the United States.^12^ Predominantly peer-run and informed by a 12-step abstinence framework, CRPs provide counseling services, recreational activities, life skills workshops, and both academic and financial support.^13^ Some provide drug-free housing on campus and typically do not have limitations on duration of stay.

There is neither an accreditation process nor a single CRP model. However, the Association of Recovery in Higher Education and Texas Tech University’s Center for Addiction and Recovery have developed guidelines for programming and implementation (https://www.depts.ttu.edu/hs/csa/replication.php). Given that these guidelines are not mandatory, CRPs differ in the way services are provided, their cost to students, and their eligibility criteria (e.g., length of abstinence, verification of abstinence). Some CRPs implement contracts that delineate behaviors to which members are expected to adhere.

Data from a national survey of 29 CRPs revealed that 57% of students are male, and 91% of students identify as White.^14^ These demographic characteristics may reflect inequitable access across diverse populations to treatment and to 4-year universities. The average age of participants was 26 years, making this group older than the average college student. The majority of the students surveyed reported drug use disorder as their primary problem and AUD as their second. Additionally, 83% of students reported having received treatment for alcohol and/or drug use prior to enrolling in the program.

To date, no national studies have evaluated the effectiveness of CRPs, but smaller-scale studies and site-specific reports show promising recovery and educational outcomes. These positive outcomes include low relapse rates, grade-point averages (GPA) above the school average, high graduation rates, and perceived usefulness by members. A survey consisting of 29 CRPs reported that annual relapse rates ranged from 0% to 25%, with an average of 8%.^14^ Additionally, only 5% of students reported using alcohol or drugs in the past month. These relapse rates are much lower compared to the first-year posttreatment relapse rates among youth.^13^ Students who participate in CRPs have higher GPAs and higher retention and graduation rates compared to national averages for the general student population.^3^

As an example, the Texas Tech University program found a semester relapse rate of 4% to 8% among participants. In addition to lower relapse rates, CRP participants at Texas Tech had a 70% graduation rate, surpassing the graduation rate of the general student population at the university.^15^ Follow-up studies on CRP alumni have found that benefits extended beyond graduation.^16^ Current findings also have implications for the recruitment of students in recovery into colleges and universities. In one study, 34% of participants surveyed expressed they would not be in college were it not for a CRP and 20% indicated that they would not have enrolled at their institution if a CRP had not been available.^17^

## RECOVERY HOMES

Recovery homes (RHs) are community-style residences open to individuals maintaining a sober lifestyle. Residents of these homes are often individuals who have undergone and exited a drug rehabilitation program or incarceration and who have entered into an RH of their own volition or by court order. All residents must avoid drugs or alcohol while living in RHs. Typically, these homes are single-sex, and residents are expected to find employment and engage in external programs—such as Alcoholics Anonymous (AA), Narcotics Anonymous, or Cocaine Anonymous—that promote their commitment to sobriety. These homes afford residents with supportive social networks of individuals also living a sober lifestyle.

RHs manifest varying intensities of structure and support for their residents, and are classified into four levels of support.^18^ Level I homes are self-run and do not include any external professional services. Level II homes often include a resident who is paid to oversee and maintain the home and to coordinate peer groups and services for residents. Level III homes often have staff present in the home who might provide clinical services and administrators who coordinate other services. Level IV homes are usually state-licensed and, as such, have licensed clinical services, are connected to state-funded services, and may be housed within a larger state-level institution.

The Washington Temperance Society started the earliest known RH in the United States in 1841.^19^ In the middle half of the 20th century, RHs expanded across the country—fostered by religious groups, state governments, and private institutions—often branching into distinct systems. For example, more than 500 houses in the Sober Living Network in Southern California are closely associated with AA. It is unclear how many RHs exist, but recent estimates suggest there may be more than 17,500 such houses in the United States.^20^

The most well-known organizations that oversee RHs are the National Alliance for Recovery Residences and the Oxford House network; of these, the latter has been more well studied. Oxford Houses are self-governed homes within the Level I designation. Responsibilities of maintaining the home, establishing and enforcing house rules, and paying rent are distributed among residents. Research on Oxford Houses suggests that residents who remain in the houses for a minimum of 6 months are significantly less likely to relapse than are those who are not provided this housing or who stay for less than 6 months.^21^ The collective and individual responsibility necessary to live at an Oxford House may motivate individuals to stay sober and provides each resident with motivated housemates who support sobriety.

Longitudinal findings from Level II homes have found that engagement in 12-step groups is the single best predictor of positive long-term outcomes for residents.^22^ When paid staff or counselors are present in the RH, as in Level III homes, residents can access psychiatric treatment and receive a structured and formalized recovery plan. Residents in these RHs, compared to individuals who enter exclusively clinical programs, have longer durations of stay and better sobriety and criminology outcomes, all at a significantly lower cost.^23^

Level IV RHs frequently house individuals who have been court-mandated to enter into a recovery program. These systems usually exist within larger institutions, are run by staff, and are known as residential therapeutic communities. Martin et al. found that, 5 years after exiting a Level IV therapeutic community, individuals who had resided in community-based therapeutic communities had lower rates of drug use and re-arrest than did those who had been in prison-based therapeutic communities, and both samples had better outcomes than individuals in the study who received no treatment.^24^

## RECOVERY COACHES

A multitude of community-based self-help groups use a mentorship-style model for recovery (e.g., sponsorship in AA). These services are provided free of cost and are typically peer-driven. The more experienced members tend to “sponsor” the newcomers,^25^ sharing their lived experiences with recovery and providing social support and access to recovery resources. Similar peer-driven recovery models are beginning to utilize recovery coaches (RCs). The first articles on RCs appeared between 1994 and 1998, coinciding with the beginning of the Recovery Community Services Program, which was instrumental in recognizing the role of peer-to-peer support services as a means of delivering treatment for drug use disorders.^26^ The reference term “recovery coach” has been evolving, from “patient navigator” to “peer recovery specialist.” Typically, RCs are peers who share their experiences of drug use and recovery with newer members and provide resources designed to build their mentees’ problem-solving abilities.^27^,^28^

RCs, who are typically in recovery themselves, are trained to provide supportive services (i.e., psychological, social, emotional, spiritual, employment, financial) to those who struggle with a substance use disorder. Employed through a variety of community groups (e.g., community centers, religious organizations), RCs generally work full- or part-time hours and are typically required to have completed high school and have earned a formal training certificate.^27^ Sharing past lived experiences with SUD and recovery cultivates trust from newcomers (who may be apprehensive about asking for help), which has been shown to increase motivation toward changing problematic behavioral patterns.^28^ Overall, RCs model recovery values of honesty and open-mindedness, a capacity for introspection, problem-solving abilities, the construction of a recovery-based identity, as well as a recovery-supportive social network.^29^

A number of factors can distinguish an AA sponsor from an RC; for example, sponsors typically work within the framework of their respective 12-step fellowship, whereas RCs offer a larger range of services and resources that fall outside of the expertise of an AA sponsor.^30^ In contrast to RCs, “recovery allies” provide the same services—that is, supporting behavior change, relationship building, harm reduction, and systems navigation^28^—but lack the “lived experience” component of an RC.^27^

Several studies have found supplemental advantages of utilizing an RC to provide recovery-specific social support. Ryan et al. found that, compared to receiving services as usual, the addition of an appointed RC significantly increased the likelihood for achieving a stable reunification for families.^31^ VanDeMark et al. found that 54% of participants endorsed RCs as helpful in creating feelings of being part of a community.^32^ Reif et al. found that RCs are effective across four domains: (1) improved relationships with providers and social supports, (2) increased treatment retention, (3) increased satisfaction with the overall treatment experience, and (4) reduced rates of relapse.^33^

## RECOVERY COMMUNITY CENTERS

Recovery community centers (RCCs) provide a variety of services such as recovery coaching, space for 12-step meetings, employment opportunities, and educational linkages. They are often located in central areas within cities and towns, with services being provided by peer volunteers and recovery professionals.^34^ RCCs do not subscribe or endorse just one ideology or pathway to recovery, but rather embrace all recovery approaches.^35^ Alcohol and drug use are reduced by providing personal, social, and environmental resources and by being flexible to multiple recovery strategies.^36^

Unfortunately, there have been few investigations of RCCs.^37^,^38^ In one of the few comprehensive investigations, Kelly et al. studied 32 RCCs across the northeastern United States.^39^ Services included social/recreational activities, mutual help, recovery coaching, employment help, education assistance, overdose reversal training, and medication-assisted treatment support. The RCCs studied were in operation for an average of 8.5 years, with considerable variability in how many clients were served each month, ranging from a dozen to more than 2,000. Most were state-funded with yearly budgets ranging from $17,000 to $760,000. Locations were primarily in urban or suburban areas with easy accessibility. The neighborhoods and buildings were rated as moderate to good in attractiveness and quality. Most but not all directors and staff were in recovery themselves.

Kelly et al. also interviewed more than 300 clients attending these RCCs.^40^ With an average age of 41, about 50% of participants were female, 79% were White, 49% had a high school or lower education, and 45% had a household income of less than $10,000 over the past year. Their primary substance of use was opioids or alcohol, and 49% reported a lifetime psychiatric diagnosis. The investigators found that the RCCs were associated with increased recovery capital (the sum of personal and social resources that facilitate the process of recovery), and that recovery capital and social support were related to improvements in psychological distress, self-esteem, and quality of life.

## DISCUSSION

This article reviews various recovery support services available in the United States throughout the life span—from adolescence through adulthood. The support services reviewed include recovery high schools, collegiate recovery programs, recovery housing, recovery coaches, and recovery community centers. These types of programs are of particular importance given that alcohol and drug use disorders are chronic conditions marked by cycles of recovery, relapse, and repeated treatment.^41^ Too often, these conditions have been treated without any attention to community factors that can contribute to abstinence or relapse. These disorders should be treated like any other chronic condition, with long-term care and treatment. Effectively treating alcohol and drug use disorders requires a paradigm shift away from pathological models of recovery and toward a multidimensional recovery health framework that encompasses the environmental context.

As noted in this article, attention is increasingly focused on supportive recovery networks, along with housing and job opportunities for social reintegration. These environmental factors highlight the importance of recovery capital.^42^ The personal component of recovery capital includes endowments such as self-efficacy, knowledge, personal health, education, hope, employment, financial assets, and transport. The social/environmental component can be further subdivided into a social branch (supportive, pro-recovery relationships with family and significant others, peer mentors, and recovery and support groups), and a community branch (treatment resources and support services, social acceptance and lack of stigma, continuum of care resources, and non-SUD support services for mental and physical health).

RHS have the potential to provide a protective environment to promote and maintain recovery for adolescent youth. Students of diverse backgrounds may benefit from access to these schools. Unfortunately, the scarcity of outcome studies makes it difficult to understand the outcomes for youth attending these settings. In addition, it is still unclear if proximity to drug-using students increases the risk of relapse. Future research should examine students’ social networks to assess both positive and negative effects of attending these alternative schools. There is also a need to better understand how to increase program sustainability of these schools.

CRPs seem to help students successfully manage their recovery while they complete their education in college and university settings, environments that are often not conducive for recovery. The lack of uniformity across CRPs limits understanding of the available findings. Further research is needed to evaluate the effectiveness of CRPs in determining which services generate the best outcomes and which pre-program enrollment characteristics (e.g., length of sobriety) can optimize student outcomes. There is also a need to investigate barriers to program implementation and to understand how to improve access and delivery of CRP resources to students. More research on post-program outcomes is needed to determine the long-term effects of participation in CRPs. Furthermore, whether CRPs can be as successful for individuals from diverse racial, ethnic, gender, and socioeconomic backgrounds needs to be examined.

In regard to recovery homes, individuals who stay in an RH for at least 6 months appear to have better long-term outcomes than those who do not stay as long. However, self-selection is at work here as a potential bias, given that the outcome might be different if people were randomly assigned to receive differing lengths of stay. There is a need to identify the location and availability of recovery houses across different regions of the United States. In addition, information about whether these homes have openings for prospective residents should be made available to the public. There is also a need to better understand the underlying processes that might account for a successful or unsuccessful stay in recovery housing; this would help determine which aspects of these homes and living communities are related to long-term sobriety. Finally, oversight of RHs by organizations such as Oxford House or by state regulatory agencies could curb the potential exploitation of residents in poorly managed houses.

RCs appear to be a helpful part of the recovery support environment, but there is a need to determine their unique contributions to outcomes. Regarding RCs and other types of recovery support services, developing a commonly agreed upon set of outcome measures in studies could advance the research in this area.^43^ This could occur with oversight committees to encourage agreements from critical stakeholder groups (e.g., outreach workers, hospitals, outpatient clinics, inpatient treatment centers, RHs).

Findings from RCCs suggest that they may facilitate the acquisition of recovery capital and enhance functioning and quality of life. It appears that individuals with few resources make use of these accessible RCCs, which may increase social support, employment, housing, and other recovery capital. Given the spread of these RCCs over the past few years, more information is needed about the costs and benefits of these innovative settings, which may play an important factor in reducing relapse.

There are a number of limitations of the studies reviewed in this article. For example, there is an overemphasis on “smaller-scale” studies and “site-specific reports,” which can be biased in favor of the particular modality and/or site being evaluated. For example, among residents of RHs, engagement in 12-step groups was the single best predictor of positive outcomes, but these types of outcomes could be biased by the self-selection of individual clients into 12-step engagement and may not indicate any additional benefit of the housing. Thus, it is important to sort out the effects of the particular intervention under review from the effects of ancillary services received in the setting. In addition, there are very few longitudinal studies evaluating recovery support services. Additional studies are needed to assess whether short-term sobriety gains and other observed outcomes are maintained over time. It is still unclear what mechanisms are involved in how recovery support services may help reduce relapse risk and foster stabilization and recovery; it is likely that this occurs by increasing recovery capital, but this is an area where more research is needed. Lastly, most of the studies reviewed had a predominantly White sample, thus warranting an examination of whether these recovery support services can help diverse racial or ethnic populations initiate and maintain long-term recovery.

Alcohol and drug use treatment programs have begun providing briefer formal programs followed by “aftercare,” which is sometimes a referral to AA or Narcotics Anonymous and an expectation to refrain from alcohol and drug use. Following release from a few weeks of acute treatment, follow-up stays in supportive, cohesive posttreatment settings encourage personal transformation and have been shown to reduce relapse rates.^44^ Environmental factors may be key contributors to long-term abstinence. Unfortunately, many youth and adults are not provided the types of environmental supports needed to solidify and support their recovery. There is a need to better understand possible improvements in long-term recovery outcomes for those provided these types of supports, as well as to gain information regarding their accessibility, availability, and affordability.^45^ There is also a need for more research in general across the spectrum of these services as well as additional research on the types of individuals for whom particular recovery support services may be most helpful, the most effective timing for introducing these services during the recovery change process, and how these services confer benefits.
